# Research on Self-Diagnosis and Self-Healing Technologies for Intelligent Fiber Optic Sensing Networks

**DOI:** 10.3390/s25061641

**Published:** 2025-03-07

**Authors:** Ruiqi Zhang, Liang Fan, Dongzhu Lu

**Affiliations:** 1 State Key Laboratory of Advanced Marine Materials, Institute of Oceanology, Chinese Academy of Sciences, Qingdao 266071, China; zhangruiqi232@mails.ucas.ac.cn; 2 University of Chinese Academy of Sciences, Beijing 100049, China

**Keywords:** fiber optic sensing network, self-diagnosis and self-healing, redundant path, optical switch

## Abstract

To address the issue of insufficient reliability of fiber optic sensing networks in complex environments, this study proposes a self-diagnosis and self-healing method based on intelligent algorithms. This method integrates redundant fiber paths and a fault detection mechanism, enabling rapid data transmission recovery through redundant paths during network faults, ensuring the stable operation of the monitoring system. Unlike traditional self-diagnosis techniques that rely on an optical time domain reflectometer, the proposed self-diagnosis algorithm utilizes data structure analysis, significantly reducing dependence on costly equipment and improving self-diagnosis efficiency. On the hardware front, a light switch driving device that does not require an external power source has been developed, expanding the application scenarios of optical switches and enhancing system adaptability and ease of operation. In the experiments, three fiber optic sensing network topologies—redundant ring structure, redundant dual-ring structure, and redundant mesh structure—are constructed for testing. The results show that the average self-diagnosis time is 0.1257 s, and the self-healing time is 0.5364 s, validating the efficiency and practicality of the proposed method. Furthermore, this study also proposes a robustness evaluation model based on sensor perception ability and coverage uniformity indicators, providing a theoretical basis for the self-healing capability of fiber optic sensing networks. This model aids in network topology optimization and fault recovery strategy design, contributing to the improvement of the stability and reliability of fiber optic sensing networks in practical applications.

## 1. Introduction

With the rapid development of optical fiber sensing technology, its applications in environmental monitoring, structural health assessment, and energy sectors are expanding significantly [1,2]. Benefiting from its excellent performance, such as high sensitivity, electromagnetic interference resistance, and long-distance data transmission, fiber optic sensors have become one of the key components of monitoring systems in complex environments [3,4,5]. However, despite the significant potential of optical fiber sensors in various application scenarios, their reliability in extreme environments remains a major challenge. Traditional individual optical fiber sensors, while capable of providing high-precision and stable measurement data, face several shortcomings when the monitoring system scales up and environments become more complex [6,7,8,9,10]. These limitations make it difficult for them to meet the monitoring needs of complex environments. In contrast, sensor networks with advanced topologies possess excellent information transmission and processing capabilities. They enable the interconnection of numerous sensors for large-scale collaborative monitoring, effectively compensating for the limitations of individual sensors and providing better solutions for monitoring in complex environments. Therefore, combining fiber optic sensors—known for their superior monitoring capabilities—with advanced topology networks to construct fiber optic sensing networks has become an emerging direction for overcoming the limitations of individual monitoring and improving monitoring efficiency [11].

In practical applications, fiber optic sensing networks often face challenges under unstable conditions or extreme environments, particularly data loss caused by network failures. Therefore, achieving self-diagnosis and self-healing capabilities for fiber optic sensing networks to ensure they can quickly restore normal operation in the event of a failure has become an important issue that needs to be addressed in the current research. Although initial progress has been made in fault detection and recovery in fiber optic sensing networks, existing studies primarily focus on improving network self-healing capabilities through topology design [12,13,14,15,16], theoretical research on self-diagnosis and self-healing [17,18,19], and sensor signal demodulation [20,21]. Chang et al. present a large-scale three-layer-ring optical fiber sensor network with reconfigurable routing functionality, achieving self-healing capabilities through optical switches and novel remote nodes [12]. Jia et al. introduce a self-healing passive FBG sensor network. Experimental validation confirms the network’s self-healing ability and determines the intensity threshold for FBG sensing signals [13]. Some studies have also developed sensing network systems with various complex structures, incorporating remote nodes and optical switches to provide strong self-healing capabilities, allowing the network to reconfigure paths and ensure stability in the event of failures. However, these studies do not provide detailed descriptions of the specific fault diagnosis and self-healing control processes, limiting the practical application and optimization of the system [14,15,16]. Hu et al. compare the repair accuracy and recovery time of three shortest path algorithms, providing valuable insights for practical engineering applications [17]. Xu et al. propose an intelligent control system that enables self-healing functionality in fiber optic sensing networks and validate its feasibility and effectiveness [18]. Another study introduces a fault resolution method and a system monitoring approach for the designed network, demonstrating its potential for large-capacity multi-parameter measurement applications [19]. In terms of demodulation, by applying different strains to FBG to collect data for training a deep learning model [20], combined with a gated recurrent unit (GRU)-based design [21], high-precision demodulation of overlapping spectra is achieved. This approach not only enhances multiplexing capacity but also significantly improves the overall performance of the fiber optic sensing network.

These approaches have significantly advanced the field by enhancing the robustness and fault tolerance of the networks. However, a key limitation of the current research is that it largely addresses these aspects in isolation, with little emphasis on integrating fault detection, diagnosis, and self-healing into a cohesive and adaptive framework. Many studies focus on theoretical models or specific network topologies, but these models often fail to consider the practical challenges associated with implementing such systems in real-world scenarios. Due to constraints such as cost, optical loss in fiber optic networks, and the complexity of large-scale systems, many of these theoretical models are difficult to realize in practice. Furthermore, most of the existing research explores self-healing mechanisms within fixed or predefined topologies, making them less adaptable to dynamic or changing network environments. At present, only a limited number of studies have integrated both fault detection and self-healing into a single framework, and even fewer have applied these integrated approaches conveniently in practical scenarios [22]. This lack of integration, flexibility, and real-world applicability continues to hinder the development of efficient, adaptable, and real-time solutions for fault detection and recovery in fiber optic sensing networks.

Research that effectively integrates self-diagnosis and self-healing mechanisms and conducts practical testing remains relatively scarce. Particularly, achieving comprehensive self-healing through the integration of hardware and software, especially by leveraging the interrelationship between self-diagnosis and self-healing, still faces numerous challenges. To address this, the present study proposes an innovative integrated self-diagnosis and self-healing mechanism, with an emphasis on upgrading and optimizing the hardware. By enhancing the synergy between hardware and software, the network becomes more efficient and stable in addressing various challenges encountered in practical applications. This innovative integration of hardware and software overcomes the limitations of existing approaches, enabling rapid fault recovery and the continuous stable operation of fiber optic sensing networks, offering a more reliable and scalable solution for practical applications.

Optical time domain reflectometer (OTDR) technology plays an important role in network diagnosis [23], but its high cost, complex operation, and stringent environmental requirements restrict its applicability in large-scale deployments, making it one of the bottlenecks in the widespread adoption of fiber optic sensing networks. Although OTDR technology has undergone years of research and development and achieves high levels of precision and accuracy [24], it is not a cost-effective choice for fiber optic sensing networks. Furthermore, OTDR only provides fault location information to support the fault detection process, without directly participating in the self-healing repair process. The design of self-healing systems for optical fiber sensing networks faces multiple challenges. One of the most critical is how to dynamically allocate system resources and coordinate network-wide monitoring to achieve seamless connections between fault detection and recovery processes [25]. In addition, in practical applications, dynamically adjusting self-healing paths may interfere with sensors that are currently functioning normally. Therefore, ensuring fault recovery while minimizing adverse impacts on other sensors remains a critical issue to be addressed [26]. For example, when a connection point between two optical fiber sensors fails, rerouting the signal through an alternative path can ensure that sensing signals from all sensors return to the demodulation system for analysis and restore the normal sensing functionality of the network. However, this dynamic adjustment may introduce interference to other normally functioning sensors, such as increased optical loss or frequent switching of optical switches [27]. These issues can compromise the stability and reliability of monitoring data, thereby limiting the widespread adoption and large-scale deployment of optical fiber sensing networks in real-world applications. To overcome these challenges, future research needs to focus on the deeper integration of hardware and software, along with seamless coordination between fault detection and recovery processes.

To address the challenges mentioned above, this study proposes a self-diagnosis and self-healing method based on intelligent algorithms, aiming to enhance the reliability and stability of the system in the event of faults. By combining redundant optical fiber paths with advanced fault detection mechanisms, this method enables the rapid restoration of data transmission through redundant paths when network failures occur. This ensures the continuous and stable operation of the monitoring system. Fault detection identifies the location of faults in the network by analyzing changes in data structures, enabling real-time fault capture and efficient recovery. Unlike traditional self-diagnosis methods that rely on OTDR, the proposed algorithm uses data structure analysis, significantly reducing the need for high-cost equipment and enhancing both efficiency and cost-effectiveness. In terms of hardware design, this study aims to develop an optical switch driver device that operates without an external power supply. This innovation addresses the power supply challenges in real-world testing environments, enhances system adaptability and ease of operation, and enables more flexible deployment of fiber optic sensing networks, especially in remote or field areas.

The structure of this paper is organized as follows: it first introduces the experimental methods and materials, then elaborates on the theoretical framework of the fiber optic sensing network model and the self-diagnosis and self-healing algorithm, providing theoretical support for the proposed method. Next, it demonstrates the implementation process and validates the effectiveness of the method. Finally, it highlights the advantages of the proposed method and summarizes the research findings.

## 2. Materials and Methods

The experimental design of this study aims to verify the effectiveness and stability of the established self-diagnosis and self-healing algorithm. To achieve this, three different fiber optic sensing network topologies are established for testing, each incorporating redundant optical fibers to enhance system robustness, ensuring that, even in the event of network failures, redundant fibers can serve as backup paths, allowing fiber optic signal data to be rapidly returned to the demodulation device (HCATM, HOSI-100B, Xi’an Huance Automation Technology Co., Ltd., Xi’an, China), ensuring the continuity and stability of the sensing network monitoring and the efficient operation of the network’s self-healing function. The main objective of this experiment is to validate the feasibility of the self-diagnosis and self-healing mechanisms, and to compare the performance differences between redundant and non-redundant networks. The self-diagnosis process involves real-time analysis of the signals collected by the demodulator to determine whether the current network state is normal. When the system detects deviations from normal operating conditions, it locates the fault point through data consistency checks, providing necessary prior information for the subsequent self-healing process. The self-healing process is initiated based on the self-diagnosis results to reconstruct the network and repair the fault. During the network reconstruction phase, the system employs a greedy algorithm to select the optimal transmission path based on the predefined topology and the status of optical switches, minimizing optical loss and ensuring network stability and signal transmission quality. The self-healing process automatically controls the switching of optical switches to restore the network and resume normal monitoring functions. In terms of network construction, this study designs three different fiber optic sensing network topologies: redundant ring structure, redundant dual-ring structure, and redundant mesh structure. These three topologies enhance the fault tolerance and stability of data transmission in the event of network failures through redundant optical fibers and multiple connection mechanisms.

The laboratory validation experiment is introduced as follows. Regarding the hardware, the optical fiber sensor used in the experiment is a fiber Bragg grating (FBG) sensor, which can sense physical quantities such as temperature and strain in real time based on its wavelength shift collected via the demodulator. In the fiber optic sensing network connection, all devices, including the optical switch, FBG, and demodulator, are connected through FC/APC fiber jumpers and flanges. The FBG model used is SMF-28, with a grating length of 10 mm, a center wavelength ranging between 1530 and 1565 nm, and reflectivity ≥95%. The fiber jumpers are fused on both sides of the FBG, and the selected flange insertion loss is ≤0.1 dB. The connection between optical switches and fiber jumpers is realized through flanges. Meanwhile, the optical switch is mounted on its drive device, and the computer system is connected to the optical switch drive device via a serial port. It is responsible for sending control commands in the event of a network failure to drive the optical switch to switch to other states and achieve self-healing of the sensing network. The fully installed fiber optic sensing network is connected to the demodulator for self-diagnosis and self-healing testing. In order to ensure that the experimental results meet the high reliability requirements in practical applications, key updates and optimizations are made to the optical switch drive device. Traditional optical switch drive devices often have disadvantages such as high cost and the need for an external power supply, which leads to inflexible installation locations. When numerous sensors are used, power supply lines may become tangled, causing confusion, making installation and problem detection difficult.

**Figure 1:**
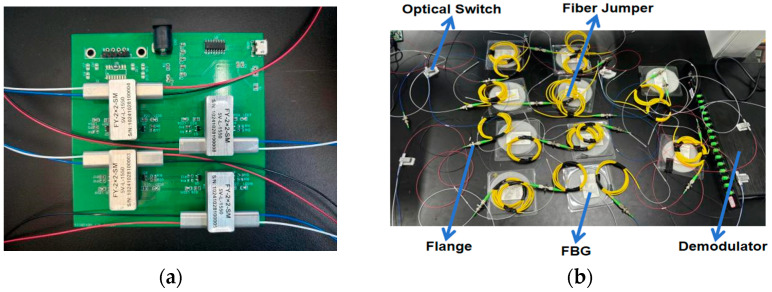
(**a**) Optical switch drive device and installation diagram; (**b**) schematic diagram of the actual network topology connection architecture.

To address this issue, this study independently develops a more practical and cost-effective optical switch drive device. This driver device operates without the need for an external power supply, designed for single-mode 1550 nm 5 V latching optical switches. The device is capable of simultaneously controlling four independent optical switches (including both 2 × 2 and 1 × 2 switches), effectively solving the problem of unstable or unavailable power in outdoor applications. The device is equipped with two 18650 standard lithium batteries, featuring low power consumption characteristics. Estimates indicate that the device can support over ten thousand optical switch switching operations. Since the network design incorporates redundant paths, the frequency of optical switch switching is kept low, preventing frequent continuous switching, thereby effectively extending the battery’s lifespan. Additionally, this innovative design significantly reduces costs while improving the convenience of the device, especially in optical fiber sensing networks that need to be deployed in complex or harsh environments. A physical image of the new optical switch drive device is shown in Figure 1a. The computer system software is connected to the drive device via a serial port. It is responsible for issuing real-time control commands to quickly switch the states of the optical switches in the event of a network failure, thereby ensuring the system can promptly recover normal operation. At the same time, the generation and sending of control commands are integrated into the self-diagnosis and self-healing algorithm. This design not only enhances the system’s self-repair capability but also improves the adaptability and stability of the fiber optic sensing network in dynamic environments.

During the experiment, manual random disconnections of the flanges are used to simulate fiber link failures. Each network topology is tested multiple times to ensure the reliability and stability of the results. Each fault simulation involves selecting a fault location randomly, recording the time taken for fault detection using the self-diagnosis algorithm, and measuring the time required for fault recovery after detection. Through these experimental designs and hardware configurations, this study not only validates the self-diagnosis and self-healing algorithm but also provides an effective solution for the practical application of fiber optic sensor networks.

## 3. Self-Diagnosis and Self-Healing of Fiber Optic Sensor Networks

### 3.1. Analysis of Self-Diagnosis and Self-Healing Algorithm

The high reliability and self-healing capability of fiber optic sensor networks are key factors in ensuring their stable operation. To address this need, this paper proposes an innovative self-diagnosis and self-healing algorithm, which combines a fault localization self-diagnosis method based on data structure analysis, and a network reconfiguration self-healing strategy that automatically switches optical switches based on diagnostic results. The goal of this method is to achieve efficient fault detection and repair, ensuring that the optical fiber sensor network can quickly recover in the event of a fault.

The self-diagnosis algorithm is the primary step in the fault detection process of optical fiber sensor networks. In the process of diagnosing optical fiber links, the signal data structure collected by the demodulator plays a crucial role. This data structure is used not only to store and manage various important parameters of the fiber optic sensors, such as optical power, signal loss, reflectivity, and wavelength, but also provides the necessary basis for real-time fault detection. The self-diagnosis algorithm primarily identifies the network status through changes in the fiber Bragg grating (FBG) signals. Under normal operating conditions, the center wavelength of each grating remains stable and is correctly detected at the receiving end. As optical signals propagate linearly along the fiber, if a fault occurs within the network, such as a fiber break or severe damage, the FBGs downstream from the fault may fail to receive optical signals, leading to data loss. Specifically, the algorithm compares the real-time collected sensing data with the preset data in the normal operating state. When the optical fiber grating signal shifts, and there are signal interruptions or abnormal fluctuations in the system, the algorithm promptly detects these deviations, and considers them indications of network abnormalities. By checking the consistency between real-time data and preset data states, the system can quickly identify potential faults, locate the fault node, and provide timely and accurate diagnostic information for the subsequent self-healing process. Through data consistency analysis, this self-diagnosis algorithm identifies the fault source in a short time, greatly improves the accuracy of fault localization, and lays the foundation for the self-healing of fiber optic sensor networks, ensuring that the system can seamlessly switch and restore normal functionality when facing anomalies.

**Figure 2:**
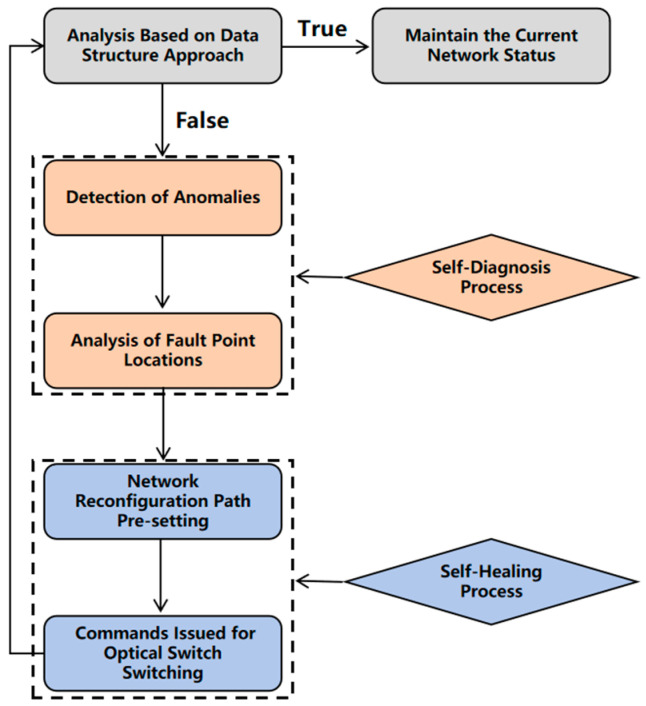
Algorithm flowchart.

After confirming the fault location, the algorithm will provide a set of network reconstruction strategies based on the topology structure, optical switch configuration, and current status to achieve self-healing. This strategy generates backup paths to bypass the faulty nodes to ensure the normal operation of the network. The preset network reconstruction plan is highly flexible, and its underlying logic design uses a greedy algorithm. Specifically, this algorithm selects the transmission path with the minimum optical loss, optimizing the signal quality and transmission stability to the maximum extent, ensuring the high transmission performance of the network after fault recovery. On this basis, the system enters the self-healing phase. At this point, according to the preset network reconstruction plan, the system sends control commands to the relevant optical switches for necessary path switching, thereby quickly restoring the data transmission function of the optical fiber sensor network. This process completes the fault repair in a short time through an efficient optical switching mechanism, avoiding prolonged system interruptions and data loss. Afterwards, the system will perform data structure analysis again to ensure that the entire optical fiber sensor network restores normal data transmission. This analysis process compares the detected data structure with the preset normal data structure to further verify the self-healing effect of the network. It then initiates normal monitoring based on the new network topology. As new connections are added, the system automatically disconnects the old (failed) paths. This ensures a rapid network restoration without disrupting the normal operation of other sensor units. The entire implementation process of the algorithm is shown in Figure 2. This algorithm achieves the complete process of fault detection, path re-planning, and self-healing repair.

### 3.2. Self-Diagnosis and Self-Healing Practical Test

In the design and implementation of the fiber optic sensing network, the primary goal is to focus on the applicability of the self-diagnosis and self-healing algorithm in real-world scenarios. Therefore, the design of the fiber optic sensing network avoids overly complex network topologies and redundant optical device configurations, simplifying the system architecture and reducing maintenance costs. On this basis, to avoid the adverse effects of dynamic path adjustments during the self-healing process on the sensors already operating normally, such as increased optical loss and frequent light switch toggling that may affect the stability of monitoring data, redundant optical fibers are introduced in the network topology design. Specifically, they are installed within the same monitoring area to form a redundant monitoring zone. The introduction of these redundant optical fibers ensures that the network can restore information transmission functionality through backup paths in the event of a fault. This design ensures that the transmission of the normal working path remains unaffected, effectively preventing instability due to extra optical loss or frequent switch toggling during fault recovery. The key to the self-healing process lies in the fact that, before a fault occurs, data are transmitted normally through the main path; once a fault occurs, the system can quickly switch to the redundant path, ensuring the stable operation of sensors within the monitoring area. Moreover, whether through the main path or the redundant path, the system ensures continuous and stable data transmission across the entire network.

**Figure 3:**
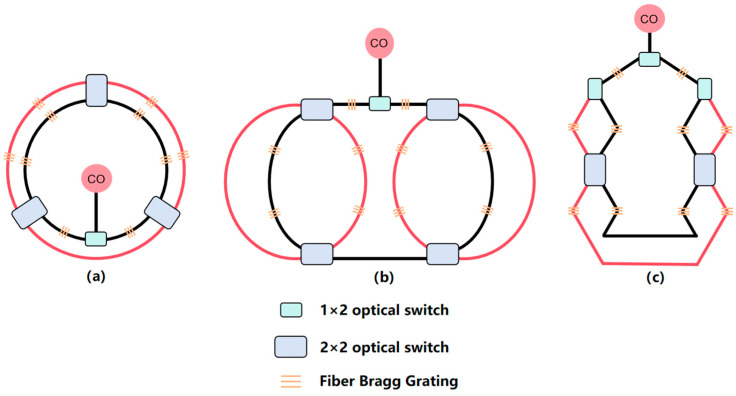
Schematic diagram of redundant topology structure: (**a**) redundant ring, (**b**) redundant dual-ring, (**c**) redundant mesh topology.

To validate the effectiveness of the proposed self-diagnosis and self-healing algorithm, this study designs and implements three types of fiber optic sensing network topologies: redundant ring topology, redundant dual-ring topology, and redundant mesh topology. Schematic diagrams of these network structures are shown in Figure 3, representing different redundancy configurations and fault recovery strategies. The redundant ring structure connects optical fiber sensors into a closed loop, allowing the network to bypass fault points through reverse transmission or redundant fibers in the event of a fault, ensuring that data transmission is uninterrupted and continues smoothly. This structure is simple and efficient, capable of rapidly recovering from faults, and avoids damage to network monitoring capabilities due to a single-point failure in the loop. The redundant dual-ring structure extends upon this by connecting each node to others with two optical fibers, forming two independent ring paths, further enhancing the network’s reliability. In the event of a fault, the network can automatically switch to the backup loop, ensuring that fault recovery does not interrupt data transmission. Compared with the redundant ring structure, the redundant dual-ring structure provides higher fault tolerance, making it suitable for applications that require higher reliability. The main advantage of the redundant mesh structure lies in its high layout flexibility and topological variability. This design not only enhances the network’s scalability and adaptability, allowing for flexible layout adjustments based on actual needs, but also effectively prevents localized faults from causing global issues, thereby improving the network’s coverage and stability. This network is particularly suitable for optical fiber sensing network applications that require rapid deployment and high adaptability. It enables efficient monitoring data transmission under various geographic and environmental conditions, ensuring long-term stable operation in complex environments. The three network topologies are shown in the figure below, where the black lines represent the normal optical signal transmission paths, and the red lines indicate the additional redundant paths.

**Figure 4:**
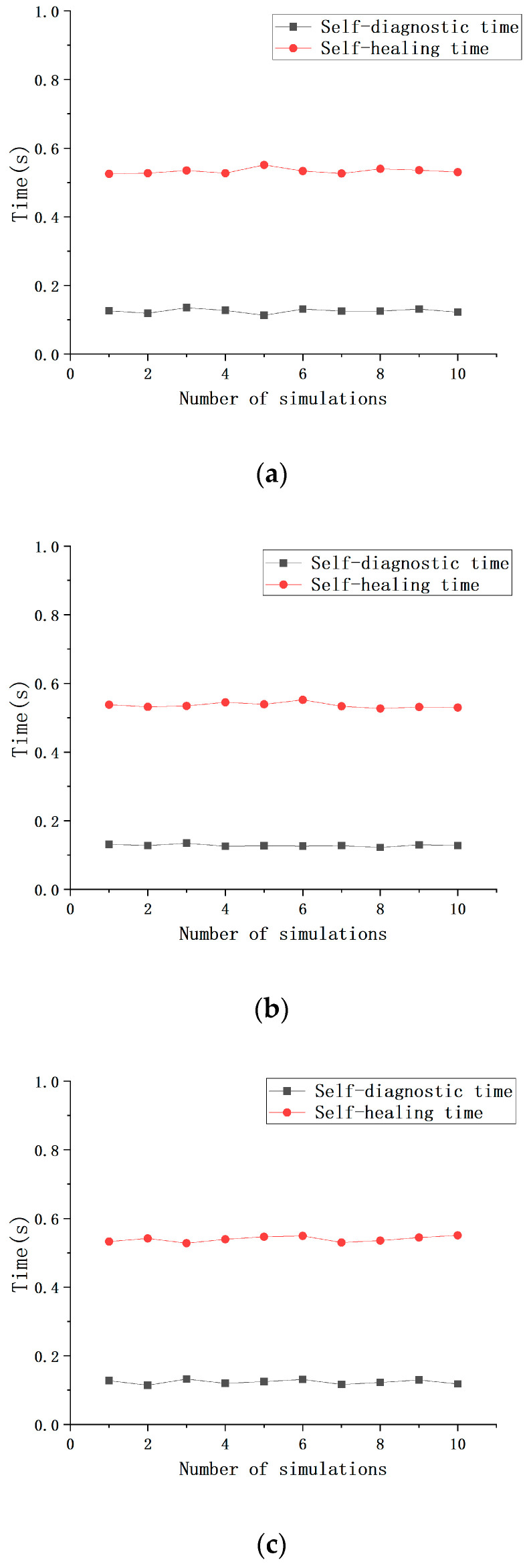
Self-diagnostic and self-healing time for three redundant networks: (**a**) redundant ring; (**b**) redundant dual-ring; (**c**) redundant mesh topology.

To validate the applicability of the proposed algorithm for self-diagnosis and self-healing in different network structures, this study conducts practical experimental tests on three network topologies. First, each fiber path in the network, including both primary and redundant paths, is numbered according to the topology structure. This ensures that each node and fiber link has a unique identifier, providing a basis for fault simulation and accurate data analysis. In the fault simulation process, a random number generator is used to randomly select 10 fault nodes, with fault occurrences restricted to the primary transmission path. Specifically, the generated random numbers determine the fault occurrence at specific nodes in the fiber link. In this experiment, faults are simulated by manually inserting and removing fiber optic jumpers. Specifically, human intervention is applied to the jumpers connected to the flange (such as insertion, removal, or partial loosening) to replicate scenarios of fiber optic fractures or poor contact, with the exact fault locations being recorded. Each network topology is tested 10 times to ensure the reliability and accuracy of the results, providing sufficient experimental data to validate the effectiveness of the self-diagnosis and self-healing algorithm. In the experiment, the following two key time metrics are defined: self-diagnosis time and self-healing time. Self-diagnosis time refers to the time interval from the occurrence of the fault to the detection of the fault by the system, while self-healing time refers to the time from the fault detection to the repair and restoration of normal operation. As shown in Figure 4, the experimental results indicate that the performance of the three network structures in the self-diagnosis and self-healing process is relatively consistent, with an average self-diagnosis time of 0.1257 s and a standard deviation of 0.0056 s. The average self-healing time is 0.5364 s with a standard deviation of 0.00796 s. The experimental results demonstrate that the proposed algorithm is capable of completing fault detection and repair in a very short time period across different topology structures, proving its efficiency and practicality. This performance provides assurance for real-time fault detection and recovery in fiber optic sensing networks, ensuring the continuous and stable operation of the system.

The simulated fault results of the three network topologies mentioned above indicate that the algorithm can rapidly identify and locate fault nodes in a short time after a system failure, providing accurate preliminary information for subsequent network reconstruction and self-healing. In contrast, the self-healing time is relatively longer, mainly due to the fact that the self-healing process not only involves fault repair but also the verification of the repair effectiveness. During the self-healing process, the system reconstructs the network path by controlling the switching of optical switches. In order to ensure that the optical switch fully switches to the predetermined state before verifying the effect, a time delay of 0.1 to 0.2 s is added to the algorithm to avoid system errors or deadlocks caused by premature verification. In more complex working environments or application scenarios that require higher monitoring stability, it may be necessary to replace the 2 × 2 optical switch structure shown in the figure with a configuration where two 2 × 2 optical switches are connected in series. Originally, a single 2 × 2 optical switch can only perform simple switching between two inputs and two outputs, while two connected 2 × 2 optical switches can achieve more complex optical signal routing. It is primarily suitable for high-fault risk environments, where it can enhance the network’s recovery capability in the event of multiple faults. The introduction of this structure can further enhance the system’s fault tolerance, ensuring that even in extreme cases, the monitoring information of the sensor network remains unaffected, and data transmission can proceed stably, even if multiple faults occur in the network. Through this redundant design, the reliability of the system can be significantly improved, providing effective assurance for meeting more demanding application requirements.

**Figure 5:**
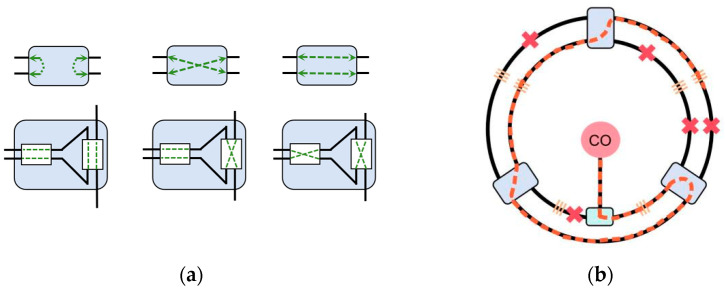
(**a**) Schematic diagram of optical switch combinations; (**b**) repair path under extreme damage.

A self-diagnosis and self-healing test is conducted on the topology network under extreme damage conditions, as shown in Figure 5. Five fault locations occur in the sensor network. Due to numerous fault points, faults need to be cleared step by step, requiring multiple optical switch operations to identify the fault status of subsequent nodes. After multiple experiments, the self-diagnosis time is approximately 1.39 s, and the self-healing time is approximately 1.91 s. In complex or high-failure risk environments, the probability of fiber optic damage is higher. This solution enhances the network’s self-healing capability, making it a viable strategy for high-risk environments.

### 3.3. Robustness Evaluation Formula for Fiber Optic Sensing Networks with Redundant Structures

With the application of intelligent fiber optic sensing networks in fields such as marine monitoring and environmental surveillance, the robustness of the network (its ability to maintain functionality and stability in the face of various faults and challenges) has become a key indicator for measuring system reliability. In the topological networks involved in this study, sensor deployment is typically designed with redundancy to ensure that the network can continue to operate normally even if some of the connecting optical fibers fail. While the redundancy design can theoretically enhance the reliability of the network, factors such as optical fiber failures and environmental changes can still affect the network’s perception capabilities and coverage range during actual operation. Therefore, this study references several robustness evaluation models [28,29] and proposes a new robustness evaluation model based on sensor perception capability and coverage uniformity. This model analyzes various network states and provides a quantitative assessment of network robustness.

To effectively describe the sensor’s perception capability at the monitoring point, a mathematical model is proposed, considering sensor status, environmental factors, and transmission attenuation. Let the perception capability of sensor *i* at time t for monitoring point d be denoted as:

(1) $$F_{i}\left(r,t\right)=\varepsilon \left(t\right)\times S_{i}\times e^{-\alpha d_{ir}}$$

where the environmental factor ε(t) varies with time and represents the impact of external environmental conditions (such as temperature, humidity, and atmospheric pressure) on the sensor’s monitoring capability. The variation of the environmental factor is typically obtained through experiments or long-term monitoring data, and it exhibits significant temporal correlation. $S_{i}$ represents the sensor’s operational status, where a value of 1 indicates the sensor is operating normally, and a value of 0 indicates a sensor fault or failure. This status can be detected in real time through the self-diagnosis algorithm established in this study. α is the signal attenuation coefficient, describing the signal attenuation of the sensor with distance, typically determined by experimental data or the optical material properties. $d_{ir}$ is the straight-line distance between sensor *i* and monitoring point *r*. This model comprehensively considers the sensor’s position and the influence of environmental changes, providing a comprehensive quantitative basis for calculating the monitoring point’s perception capability. The total coverage capability C(r,t) of monitoring point *r* is given by the sum of the perception capabilities of all sensors in the network. The formula can be expressed as:

(2) $$C\left(r,t\right)=\sum_{i=0}^{n}F_{i}\left(r,t\right)$$

where *n* represents the total number of sensors in the network. This formula provides a comprehensive assessment of how point r is sensed by all the sensors in the network. To further assess the uniformity of network coverage, the following two key metrics are introduced: mean coverage capability and coverage capability variance. The mean coverage capability represents the average coverage ability of all monitoring points in monitoring area *D*, and the formula for calculating it is:

(3) $$G_{k}=\frac{1}{\left|D\right|}\sum_{r\in D}C\left(r,t\right)$$

This metric reflects the overall coverage level within the area. If $G_{k}$ is high, it indicates that the overall coverage capability of the monitoring area is high. The coverage ability variance describes the degree of dispersion in the coverage ability within the monitoring area. The formula is:

(4) $$Var\left(C_{k}\right)=\frac{1}{\left|D\right|}\sum_{r\in D}\left[C\left(r,t\right)-{G_{k}}^{2}\right]$$

This variance reflects the uniformity of coverage within the area. A larger variance indicates uneven coverage, which may result in weaker system robustness when facing faults; a smaller variance indicates uniform coverage, suggesting higher system robustness. The failure probability of the network state $P(S_{k})$ is calculated based on the failure probability $P_{i}$ of the sensors and the failure correlation coefficient $f_{ij}$ between the sensors, which is caused by the damage to the connecting optical fibers. The formula is introduced as follows:

(5) $$P\left(S_{k}\right)=\prod_{i=1}^{n}\left[{P_{i}}^{1-S_{i}}{\left(1-P_{i}\right)}^{S_{i}}\right]\times \prod_{i\neq j}\left[{\left(1+f_{ij}\right)}^{S_{i}}\right]$$

$f_{ij}$ represents the increment in the fault signal transmission of sensor *j* when sensor *i* fails. Due to the redundancy added in the fiber optic sensing network used in this study, the working fiber Bragg gratings and redundant gratings are located relatively close to each other, which may be affected by environmental factors in the same area or suffer from localized damage. This formula allows for a comprehensive consideration of the impact of network failures on robustness, providing a more accurate basis for robustness evaluation. The final network robustness R is comprehensively evaluated by considering the mean coverage ability and the coverage ability variance of the network. Assuming that the network state $S_{k}$ consists of the operational states of all sensors, the network robustness can be calculated using the following formula:

(6) $$R=\sum_{k=1}^{2^{n}}P\left(S_{k}\right)\times \left[\frac{G_{k}}{1+Var\left(C_{k}\right)}\right]$$

$P(S_{k})$ represents the probability of the network being in state $S_{k}$, $G_{k}$ is the mean coverage ability in that state, and $Var(C_{k})$ is the coverage ability variance in that state.

**Figure 6:**
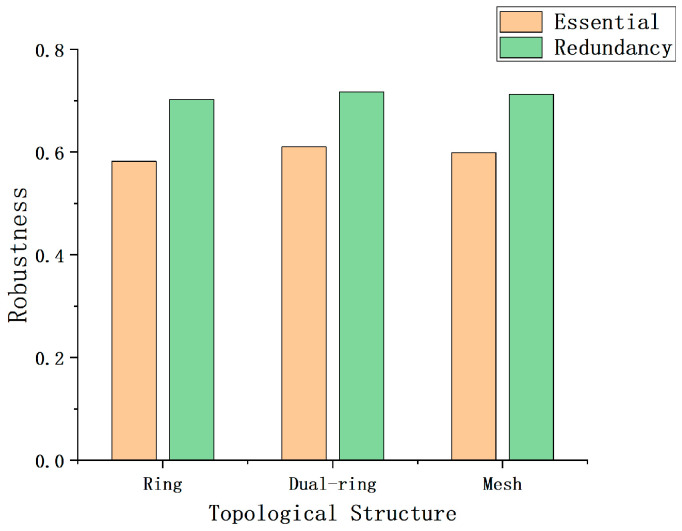
Robustness values of the three redundant networks and their non-redundant counterparts.

In order to explore the robustness of the three proposed fiber optic sensing network topologies and their corresponding non-redundant topologies, this study performs numerical simulations and evaluates them based on the robustness assessment model presented earlier. First, reasonable value ranges are set for the parameters involved in the formulas. Specifically, according to the relevant literature and industry reports, the expected lifetime of optical fiber sensors can exceed 20 years. Therefore, it is assumed that the environmental factor coefficient ε(t) of the optical fiber sensor decreases exponentially over time, starting at 1 in the first year and gradually reducing to 0 by the 20th year. However, since this experiment focuses on the robustness evaluation during the early stages of network construction, the performance degradation coefficient of the optical fiber sensors is set to 1 in the calculations to simplify the simulation. Secondly, since all the sensor models and performance within the network are identical in the experiment, the attenuation coefficient α of the sensors is set to 0.05 [28], and both the sensor failure probability and the influence increment of sensors in the redundant area are set to 0.2 [28]. It should be noted that these values are only used for the comparison of network robustness in the simulation experiments, and the specific parameters in practical applications need to be adjusted according to the actual test results of the network. In the numerical simulation process, the simulation area is a 100 × 100 two-dimensional monitoring region, and 10,000 monitoring points are randomly selected for the robustness test. In the simulation process, the number of FBGs is kept consistent across different structures to ensure comparability of the results. By comparing and analyzing the robustness of three different network topologies and their corresponding non-redundant topologies, the simulation results shown in Figure 6 are obtained. The experimental results indicate that all redundant networks exhibit a significant improvement in robustness.

## 4. Conclusions

This paper proposes a self-diagnosis and self-healing algorithm based on data structure analysis and optical switch control, aimed at improving the reliability and stability of fiber optic sensing networks under complex operating conditions. By combining redundant optical fiber design and the optimization of optical switch control strategies, three network topologies—redundant ring structure, dual-ring structure, and mesh structure—are designed and experimentally validated.

The system demonstrates high efficiency and consistency in fault detection and repair. Experimental results show that the average self-diagnosis time for the three network structures is 0.1257 s, with a standard deviation of 0.0056 s; the average self-healing time is 0.5364 s, with a standard deviation of 0.00796 s. These results indicate that the system can rapidly localize faults and repair the network within a very short time. The low standard deviations further demonstrate the system’s high stability across multiple trials, with consistent fault diagnosis and repair outcomes.

This study proposes a robustness evaluation model based on sensor perception capability and coverage uniformity indicators, providing a quantitative method for assessing the robustness of sensing networks. This model not only provides a theoretical basis for evaluating the self-healing capability of fiber optic sensing networks but also offers systematic support for optimizing network topology and designing fault recovery strategies.

## 5. Future Work

To further enhance the convenience and adaptability of the sensing network, improvements are needed in the driver board design, specifically by integrating WiFi or Bluetooth control modules. The enhancement will allow users to operate the device independently via wireless methods, eliminating the need for a computer. As a result, the control of fiber optic sensing networks will become more flexible and efficient. Through encapsulation and optimization, the driver device can meet the demands of a broader range of practical scenarios, significantly enhancing the system’s scalability and versatility.

Future research will focus on optimizing the optical switch control strategy and enhancing the algorithm response speed to reduce the self-healing time. This will improve the system’s adaptability and performance in more complex environments.

## Data Availability

All relevant data will be made available upon request.
